# Increasing the uptake of Intermittent Preventive Treatment of malaria in pregnancy using Sulfadoxine-Pyrimethamine (IPTp-SP) through seasonal malaria chemoprevention channel delivery: protocol of a multicenter cluster randomized implementation trial in Mali and Burkina Faso

**DOI:** 10.1186/s12889-023-17529-z

**Published:** 2024-01-02

**Authors:** Kadiatou Koita, Joel D. Bognini, Efundem Agboraw, Mahamadou Dembélé, Seydou Yabré, Biébo Bihoun, Oumou Coulibaly, Hamidou Niangaly, Jean-Batiste N’Takpé, Maia Lesosky, Dario Scaramuzzi, Eve Worrall, Jenny Hill, Valérie Briand, Halidou Tinto, Kassoum Kayentao

**Affiliations:** 1 Department of Epidemiology of Parasitic Diseases (DEAP), Faculty of Medicine Odontostomatology, University of Sciences Techniques and Technologies of Bamako, Bamako, Mali; 2 Institut de Recherche en Sciences de La Santé (IRSS), Unité de Recherche Clinique de Nanoro, Ouagadougou, Burkina Faso; 3 Liverpool School of Tropical Medicine, Vector Biology, Liverpool, UK; 4 Département Etudes, Institut National de Santé Publique, Recherches Médicale Et Communautaire, Bamako, Mali; 5 University of Bordeaux, National Institute for Health and Medical Research, Bordeaux, France; 6 Department of Clinical Sciences, Liverpool School of Tropical Medicine, Liverpool, UK; 7 R-Evolution Worldwide Srl Impresa Sociale (REvoWWIS), Naples, Italy; 8 University of Bordeaux, National Institute for Health and Medical Research (INSERM) UMR 1219, Research Institute for Sustainable Development (IRD) EMR 271, Bordeaux Population Health Centre, Bordeaux, France

**Keywords:** Malaria, Pregnant women, Women with a child less than 12 months of age, Tropical medicine, Infectious diseases, Maternal and child health, Epidemiology

## Abstract

**Background:**

The uptake of Intermittent Preventive Treatment of malaria in pregnancy using Sulfadoxine-Pyrimethamine (IPTp-SP) remains unacceptably low, with more than two-thirds of pregnant women in sub-Saharan Africa still not accessing the three or more doses recommended by the World Health Organisation (WHO). In contrast, the coverage of Seasonal Malaria Chemoprevention (SMC), a more recent strategy recommended by the WHO for malaria prevention in children under five years living in Sahelian countries with seasonal transmission, including Mali and Burkina-Faso, is high (up to 90%). We hypothesized that IPTp-SP delivery to pregnant women through SMC alongside antenatal care (ANC) will increase IPTp-SP coverage, boost ANC attendance, and increase public health impact. This protocol describes the approach to assess acceptability, feasibility, effectiveness, and cost-effectiveness of the integrated strategy.

**Methods and analysis:**

This is a multicentre, cluster-randomized, implementation trial of IPTp-SP delivery through ANC + SMC vs ANC alone in 40 health facilities and their catchment populations (20 clusters per arm). The intervention will consist of monthly administration of IPTp-SP through four monthly rounds of SMC during the malaria transmission season (July to October), for two consecutive years. Effectiveness of the strategy to increase coverage of three or more doses of IPTp-SP (IPTp3 +) will be assessed using household surveys and ANC exit interviews. Statistical analysis of IPT3 + and four or more ANC uptake will use a generalized linear mixed model. Feasibility and acceptability will be assessed through in-depth interviews and focus group discussions with health workers, pregnant women, and women with a child < 12 months.

**Discussion:**

This multicentre cluster randomized implementation trial powered to detect a 45% and 22% increase in IPTp-SP3 + uptake in Mali and Burkina-Faso, respectively, will generate evidence on the feasibility, acceptability, effectiveness, and cost-effectiveness of IPTp-SP delivered through the ANC + SMC channel. The intervention is designed to facilitate scalability and translation into policy by leveraging existing resources, while strengthening local capacities in research, health, and community institutions. Findings will inform the local national malaria control policies.

**Trial registration:**

Retrospectively registered on August 11th, 2022; registration # PACTR202208844472053.

Protocol v4.0 dated September 04, 2023.

Trail sponsor: University of Sciences Techniques and Technologies of Bamako (USTTB), Mali.

## Background

Malaria infection during pregnancy is a major public health problem, and a notable cause of adverse birth outcomes, including foetal loss, stillbirth, low birth weight and preterm birth [1]. In 2021, 32% of the estimated 40 million pregnancies were exposed to malaria infection in Sub-Saharan Africa (SSA), which would have resulted in 961,000 low birth weight (LBW) children with no pregnancy-specific intervention [2]. To overcome the latter, the World Health Organization (WHO) recommends the use of intermittent preventive treatment with sulfadoxine-pyrimethamine to prevent malaria in pregnancy (IPTp-SP) in areas of moderate-to-high transmission in SSA [3]. However, IPTp-SP uptake remains unacceptably low [2]. Indeed, among the approximately 840 million people at risk of malaria in endemic countries in SSA, more than 30 million pregnant women could benefit from IPTp-SP each year. Still, during the last few years, WHO observed a decline in its coverage in several African countries. Indeed over two-thirds of pregnant women in SSA are still not accessing to the WHO-recommended three or more doses (IPTp3 +), and closing the gaps in access to proven malaria control tools is a top priority for the WHO Global Malaria Program [4].

Despite the implementation of this policy, in 2018 about 28% to 40% of pregnant women in Mali received IPTp3 + [5] when in Burkina Faso this figure was 58% [5]. In contrast, the coverage of Seasonal Malaria Chemoprevention (SMC) among children under 5 years in both countries and in other Sahelian countries of West Africa is high (up to 90%) [5]. SMC is a relatively new strategy recommended by the WHO in 2012 for malaria control in Sahelian countries with seasonal malaria transmission such as Mali and Burkina Faso. It consists of the administration of a single dose of SP plus three daily administrations of amodiaquine (SP + 3AQ) to children aged 3–59 months during the high malaria transmission season, involving four rounds of SP + 3AQ at monthly intervals. SMC is provided at fixed or door-to- door delivery (DDD) visits by community health workers (CHWs) or community relays (CRs). In the two countries, and in other Sahelian countries, both fixed and DDD strategies provide good coverage though DDD provides higher coverage [6].

Thus, we hypothesized that IPTp-SP delivery to pregnant women through the SMC channel using DDD approach will increase IPTp-SP coverage and achieve greater public health impact, as the risk of malaria and its burden on birth outcomes increases during the rainy season corresponding also to the period of SMC [7, 8]. Reported increases in two or more doses (IPTp2 +) and IPTp3 + coverage with community-based IPTp-SP administration vary considerably by country and baseline IPTp-SP coverage [9–14]. Recently, in Burkina Faso, IPTp-SP administration by CHWs (outside the SMC distribution channel) resulted in a relative increase of 17.6% in IPTp3 uptake [9]. In addition the IPTp-SP coverage has lagged behind antenatal care (ANC) coverage for years, and the integrated strategy has the potential to substantially boost it. Indeed, by giving SP in the community through CHWs and CRs during the high malaria transmission season when ANC access is reduced, whilst also mobilizing pregnant women to attend ANC, the strategy may also boost ANC attendance and uptake of the latest WHO ANC recommendations of eight ANC contacts during pregnancy [15].

The COVID-19 pandemic also posed important considerations for health care systems and service delivery in SSA. This study will explore strategies to address the likely decrease in the uptake of ANC services in the study population because of the COVID-19.

## Methods/Designs

### Objectives and hypothesis

The main objective of this study is to evaluate whether the integration of IPTp-SP delivery to SMC in addition to ANC delivery will increase the coverage of IPTp-SP and ANC among pregnant women in Mali and Burkina Faso. The primary hypothesis is that integrated ANC + SMC strategy (intervention) as compared to the ANC (standard of care) will increase coverage defined as an increase in coverage of ‘at least 3 doses’ of IPTp-SP, by 45% in Mali and 22% in Burkina Faso, and support the goal of improving the prevention of malaria in pregnancy and reducing adverse birth outcomes in Sahelian pregnant women.

The primary objectives are: (1) to compare the delivery of IPTp-SP through the integrated strategy with standard of care, (2) to assess the acceptability and feasibility among health providers and pregnant women, (3) to assess the systems effectiveness at implementing the integrated delivery strategy and (4) to estimate the incremental cost-effectiveness of the integrated versus standard of care strategy from the societal perspective. (Economic protocol to be published separately).

The secondary objectives are: (1) to assess any increase or decrease in SMC or ANC uptake following the integrated ANC + SMC IPTp-SP implementation, (2) to estimate the number of malaria cases in both children and pregnant women over the course of the trial, and (3) to assess pregnancy outcomes at delivery (birth weight, miscarriage, stillbirth, preterm birth) in both the intervention and control arms among women delivering at health facilities (HF).

An exploratory objective will assess women’s ANC and IPTp-SP care seeking practices and any impact that the COVID-19 pandemic had on their delivery and access.

### Study design

**Fig. 1:**
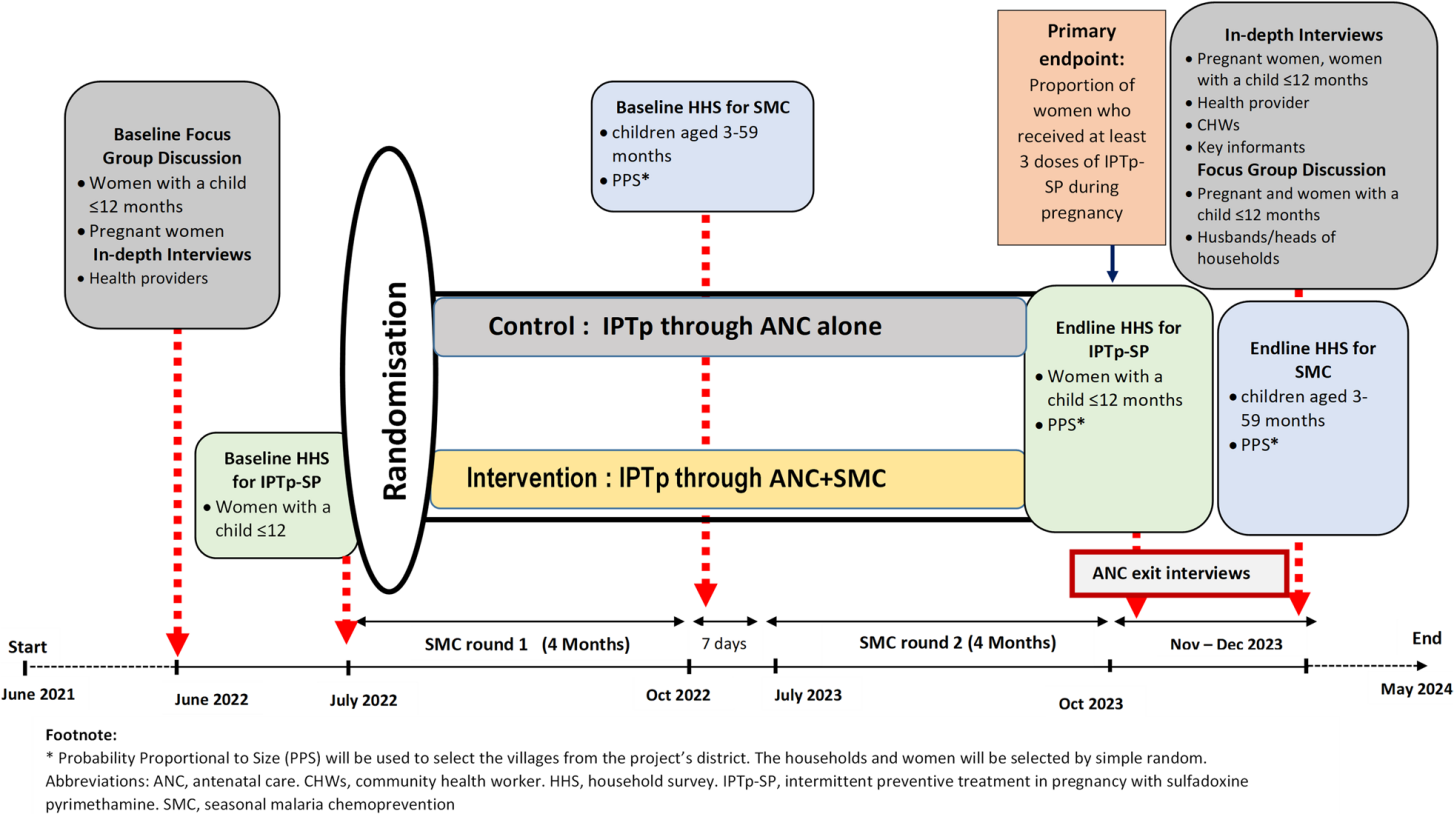
Study design and timeline

This is a multicenter, cluster randomized, implementation trial of IPTp-SP delivery in two parallel arms (ANC alone and ANC + SMC) at 40 HFs and their catchment communities (20 clusters per arm). The design and the data collection timelines are illustrated in Fig. 1.

#### Study population

Pregnant women and women with a child under 12 months of age, healthcare providers, Community Health Workers (CHWs) and or Community Relays (CRs), District Health Management Team staff.

#### Inclusion criteria

- Clusters: Government and community owned HFs.
- Participants living in the catchment area of selected government and community HFs in the study sites/clusters.
- Participants’ written informed consent.

#### Exclusion criteria

- Private HFs, ANC attendees of private HFs and staff of private HFs.

#### Sampling methods

**Fig. 2:**
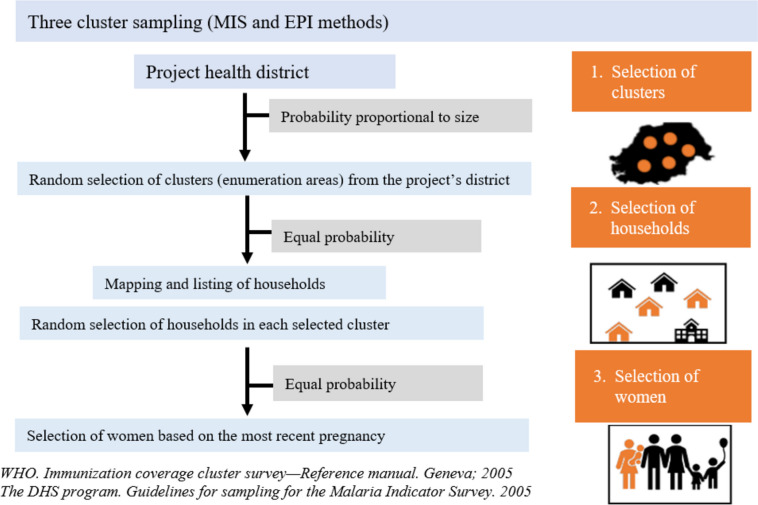
Sampling methods

Four household surveys (HHS) will be carried out. Two at baseline, one to assess participants’ baseline characteristics, IPTp-SP coverage, and ANC attendance rate, and one for SMC coverage. Two at endline, one to measure the primary endpoint (i.e., IPTp3 + coverage) and one to measure SMC coverage. In each country, a multi-stage cluster sampling method will be followed to select the clusters (health facility and its catchment area) from the study sites that will be included in the HHS. The sampling will be carried out in three stages in each study site (Fig. 2), namely: (1) random selection of ‘clusters’ using probability proportional to size (PPS); (2) random selection of households in each ‘cluster’ using maps and household listing. Mapping and listing of the households will be completed before the baseline IPTp-SP or SMC HHS begins. These households will be selected by equal probability; (3) simple random selection of the woman or guardians of SMC children to be interviewed in each household among those meeting the inclusion criteria. A single child will be selected per HH to minimize the cluster effect. When several eligible women are present in a household, the woman with the most recent birth will be chosen.

The same sampling methods will be used for the baseline and endline HHS, except for stage 1 (i.e., selection of ‘clusters’ by PPS) which at endline will be stratified by arm.

### Study site

**Fig. 3:**
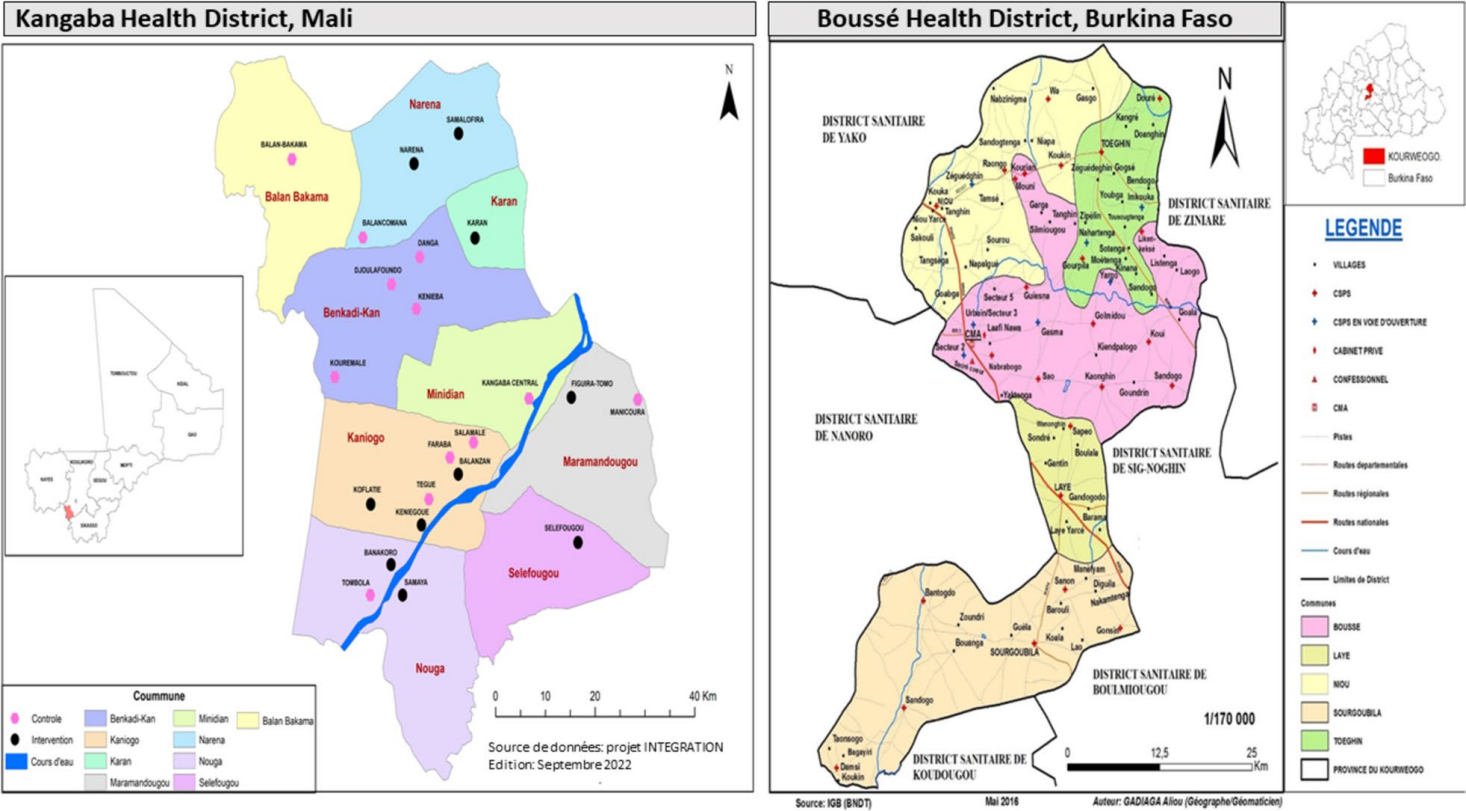
Study sites

The study is conducted in two Sahelian countries with highly seasonal malaria transmission where SMC has been implemented since 2012 (Fig. 3). In Burkina Faso, the study site is Boussé District located at 55 kms North-East from Ouagadougou. The population in 2019 was estimated at 189,937 inhabitants [16]. In 2020, the coverage of IPTp3 + and SMC was 55.9% and 103%, respectively [17]. In Mali, the study site is the district of Kangaba located at 85 kms South-West of Bamako with an estimated population of 146,563 inhabitants in 2021. The district belongs to the region of Koulikoro which has 33.8% coverage of IPTp3 + [18] and that of SMC at > 100% in the district [19].

### Randomization and treatment allocation

Randomization will be done prior to the baseline survey but kept concealed until the completion of the baseline survey. Clusters will be randomly allocated at a ratio of 1:1 to the intervention and control arms. The unit of randomization will be HF and its catchment area to avoid contamination between ANC providers in the same service. Therefore, each HF and its respective community will form a cluster. Each HF will be randomly assigned to either:

1) the delivery of IPTp-SP to pregnant women during SMC implementation + ANC (intervention); or 2) delivery of IPTp-SP using standard ANC delivery (control).

SP will be provided by the study in both intervention and control sites to avoid stock-outs. To minimize any risk of allocation bias, all participating HFs will be randomized simultaneously.

- Intervention arm

**Fig. 4:**
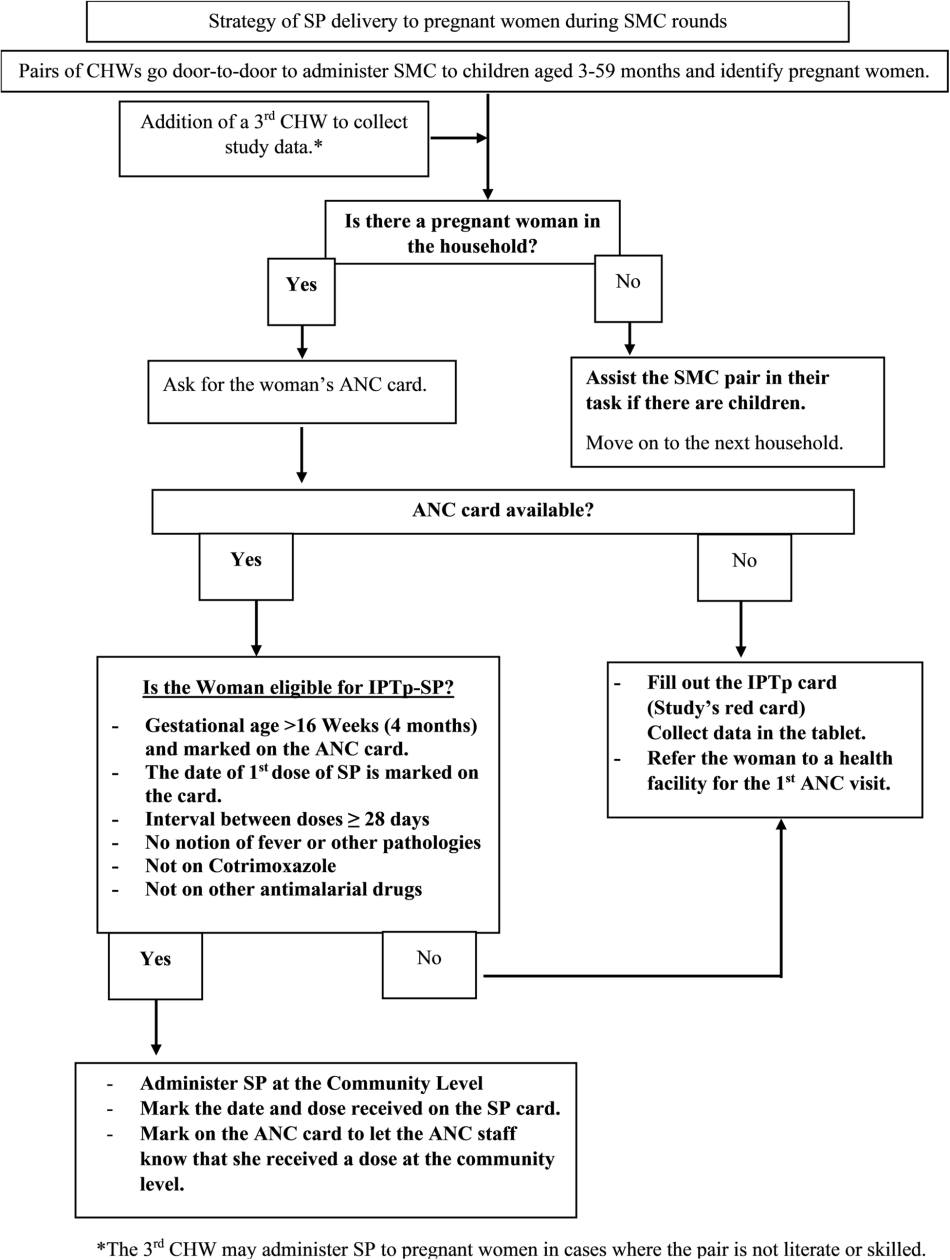
Intervention

The intervention will consist of the monthly provision of IPTp-SP through the SMC channel during the four monthly rounds (July to October) for two consecutive years (Fig. 4).

Women will be encouraged/sensitised to continue with their scheduled ANC visits to receive other essential ANC services in addition to IPTp-SP doses in between the annual SMC cycles.

- Control arm

Women will receive monthly IPTp-SP doses through scheduled ANC visits (standard care) only.

### Sample size calculation

#### Primary endpoint – IPTp3 + at endline

The primary endpoint will be collected at endline among women with a child less than 12 months.. It will be the individual level binary indicator for presence or absence of at least three doses of IPTp-SP through ANC alone or ANC plus SMC delivery platforms. Since baseline prevalence of IPTp-SP coverage highly differs between Mali and Burkina Faso, the sample size was calculated for each country separately.

In Mali, with 10 clusters per arm, 48 women sampled per cluster will be required at endline to detect a 45% increase in the relative risk of women receiving at least 3 doses of IPTp-SP between control and intervention arms, with 80% power, alpha = 5%, an intra-cluster correlation coefficient (ICC) of 2.5% and 5% primary endpoint missing rate, assuming this proportion to be 28% in the control arm [20]. Based on this assumption a total of 1,008 women will be included (Shiny CRT calculator®) [21]. With this sample size, we should be able to demonstrate a 33% in the relative risk of women receiving at least 3 doses of IPTp-SP assuming this proportion to be 40% in the control arm as recently reported in an HHS conducted in a neighboring area, Bankass in Mopti region (Kayentao et al., 2022, unpublished data). Assuming that there will be at least one eligible woman in every 5 households, a total of 5,040 households will be visited.

In Burkina Faso, with 10 clusters per arm, 46 women sampled per cluster will be required at endline to detect an increase of 22% in the relative risk of women with at least 3 doses of IPTp-SP between the control and intervention arms, with 80% power, alpha = 5%, an ICC of 2.5% and 5% primary endpoint missing rate, assuming this proportion to be 58% in the control arm [18]. Based on this assumption a total of 966 women will be included. Assuming that there will be at least one eligible woman in every 5 households, a total of 4,830 households will be visited.

A possible adjustment of the number of women to be interviewed in the endline surveys will be made based on the baseline data: IPT3 + coverage; proportion of non-response and calculated ICC.

#### Sample size – IPTp-SP and SMC coverages at baseline

The baseline HHS will determine IPTp-SP coverage among women who have delivered in the previous 12 months, and SMC coverage among children living in the study districts, in the two countries.

Table 1 shows the sample size calculated for the baseline IPTp-SP and the SMC HHS based on the following equation [22]:

**Table 1:** Estimated sample size for baseline IPTp-SP and SMC HHS

Country	Outcomes		Estimated sample size	Estimated number of households to be visited^a^
**Mali**	Estimated IPTp3 +	28%, up to 40%	775	3,875
	Estimated SMC coverage 4 doses	54%	802	802
**Burkina Faso**	Estimated IPTp3 +	58%	786	3,930
	Estimated SMC coverage 4 doses	90%	290	290

^a^It is assumed that at least one woman in every five households will meet the inclusion criteria and there will be a non-response rate of 5%. Sources: MIS 2018, for Burkina Faso [18]; ACCESS SMC partnership [23]; Diawara et al. 2017 [24] and Kayentao et al. 2022, unpublished data, for Mali

$$n=\frac{DE\cdot{1.96}^2\cdot p\cdot\left(1-p\right)}{{precision}^2}$$

Where Design Effect (DE) = 2, *p* = expected IPTp3 + coverage, or the expected SMC coverage, prevalence precision =  ± 0.05 with a 95% CI. It is assumed that at least one woman in every five households, and one child in every household will meet the inclusion criteria and there will be a non-response rate of 5%.

#### Sample size – SMC coverage at endline

The endline SMC HHS will be conducted 16 months after the implementation to determine the coverage of four doses of SMC among 3–59 months old children in project districts in the two countries and test for differences in SMC coverage between control and intervention arms.

For IPTp-SP coverage at endline we expect to visit 5,040 households in Mali, and 4,830 in Burkina Faso, which we expect will be all eligible for the SMC HHS. Among them, 880 and 120 children (one per household) randomly selected in Mali and Burkina Faso respectively will give us 80% power at 5% alpha to detect a relative difference of 25% in SMC between control and intervention arms, assuming prevalence in control arm is 54% (Mali) and 90% (Burkina Faso).

#### Secondary endpoints

- Feasibility and acceptability

To assess the acceptability and feasibility of IPTp-SP delivery through the ANC + SMC strategy, a purposive sample of 40 to 80 pregnant women of different gravidities and women with a child < 12 months. per country (80 to 160 total) will be recruited at endline. An additional purposive sample of 48 to 72 women will be selected for Focus Group Discussions (FGDs). Two to four health providers purposively selected from each of the 10 intervention HFs in each country (40 to 80 total) and all consenting CHWs who were involved in the IPTp-SP community delivery will be included. The interviews will also include the DHMTs and Ministry of Health staff in the National Malaria Control and Reproductive Health departments (approx. 5 per site, *n* = 10).

- Systems effectiveness

ANC exit interviews will be conducted with pregnant women when leaving the HF at endline to assess the systems effectiveness of the IPTp-SP delivery in both arms (intervention and control). In Mali, a sample size of 699 women from 20 HF will allow the detection of an estimated 35% of women achieving the primary endpoint of IPTp3 + with a precision of ± 5%, 95% CI, and a DE of 2. In Burkina Faso, a sample size of 699 women from 20 HF will allow the detection of an estimated 65% of women achieving the primary endpoint of IPTp3 + with a precision of ± 5%, 95% CI, and a DE of 2.

- Exploratory COVID

To assess women’s ANC and IPTp-SP care seeking practices and any impact that the COVID-19 pandemic had on delivery and access at baseline, a purposive sample of 8–12 pregnant women of different gravidities and women with a child < 12 months. will be selected (80–120 women per country for a total of 160–240) for FGD, and a purposive sample of 2–3 health care providers will be selected from 10 HFs in both countries (20–30 per country for a total of 40–60) for in-depth interviews.

### Data collection

- Primary endpoints—Household surveys for IPTp-SP, SMC, and ANC

The tablet-based survey using REDCap will be in French; the surveyors will conduct the survey in the language of the participant’s choice based on the translated questionnaire and submit the responses to the tablet in French. Surveyors will not be members of the villages they survey, nor will they be members of the intervention health care delivery staff. All surveyors will be female, as the survey tool contains potentially sensitive questions (i.e., number of pregnancies, details about a potential stillbirth, miscarriage, etc.). ANC and IPTp-SP cards will be checked for concordance with women’s self-report of ANC visits and IPTp-SP doses.

Data on SMC uptake will be collected by interviewing children’s mothers/guardians and through review of children’s SMC cards one week after the last SMC round.

#### Secondary endpoints

- Feasibility and acceptability

In-depth interviews will be conducted at endline among health managers, ANC providers, CHWs, pregnant women and women with a child < 12 months., and key informants (local authorities) in both study arms to explore their perceptions and experiences with the intervention.

FGDs with pregnant women and women with a child < 12 months will be undertaken at baseline to explore women’s ANC and IPTp-SP care seeking practices and any impact that the COVID-19 pandemic had on delivery and access. FGDs with pregnant women and women with a child < 12 months., and husbands or heads of households will be conducted at endline to explore their perceptions on the feasibility and acceptability of the proposed integrated delivery strategy.

All in-depth interviews and FGDs will use topic guides and will be recorded. Audio files will be transcribed in the local language and translated into English with quality checks at each stage of transcription and translation. Transcripts will be imported to NVivo version 12 for coding. Field notes will be typed and imported into the Nvivo project for subsequent analysis.

- Exploratory COVID

In-depth interviews will be conducted at baseline among ANC providers, CHWs and pregnant women and women with a child < 12 months. to assess pregnant women’s ANC and IPTp-SP care seeking practices and any impact that the COVID-19 pandemic had on delivery and access.

- Systems effectiveness: ANC exit interview

Pregnant women will be approached as they come into the facility and asked to participate in the study. A structured questionnaire will be used to interview enrolled pregnant women as they exit ANC. Topic categories within the questionnaire will include demographics, current and past pregnancy history, reason for attendance on the day of the interview, IPTp-SP received on the day of the interview by DOT and information given by the health provider, IPTp-SP doses received during previous ANC visits or home visits by CHWs during that pregnancy, and any other medication received. ANC and IPTp-SP cards check will also be performed to assess concordance with women’s self-report.

- Process evaluation

In parallel with the main trial, we will conduct a mixed-methods process evaluation to assess whether the study intervention was implemented as intended based on the MRC guidance for process evaluation of complex interventions [25]. The process evaluation will assess the following implementation parameters: fidelity; dose; reach; context; and adaptations made to the intervention in the study context(s). Additional complementary qualitative data from interviews with participants and implementers will clarify potential mechanisms through which the intervention achieved outcomes. These insights are of value within the context of the diverse large scale effectiveness trial settings and will inform future scale-up and sustainability.

**Fig. 5:**
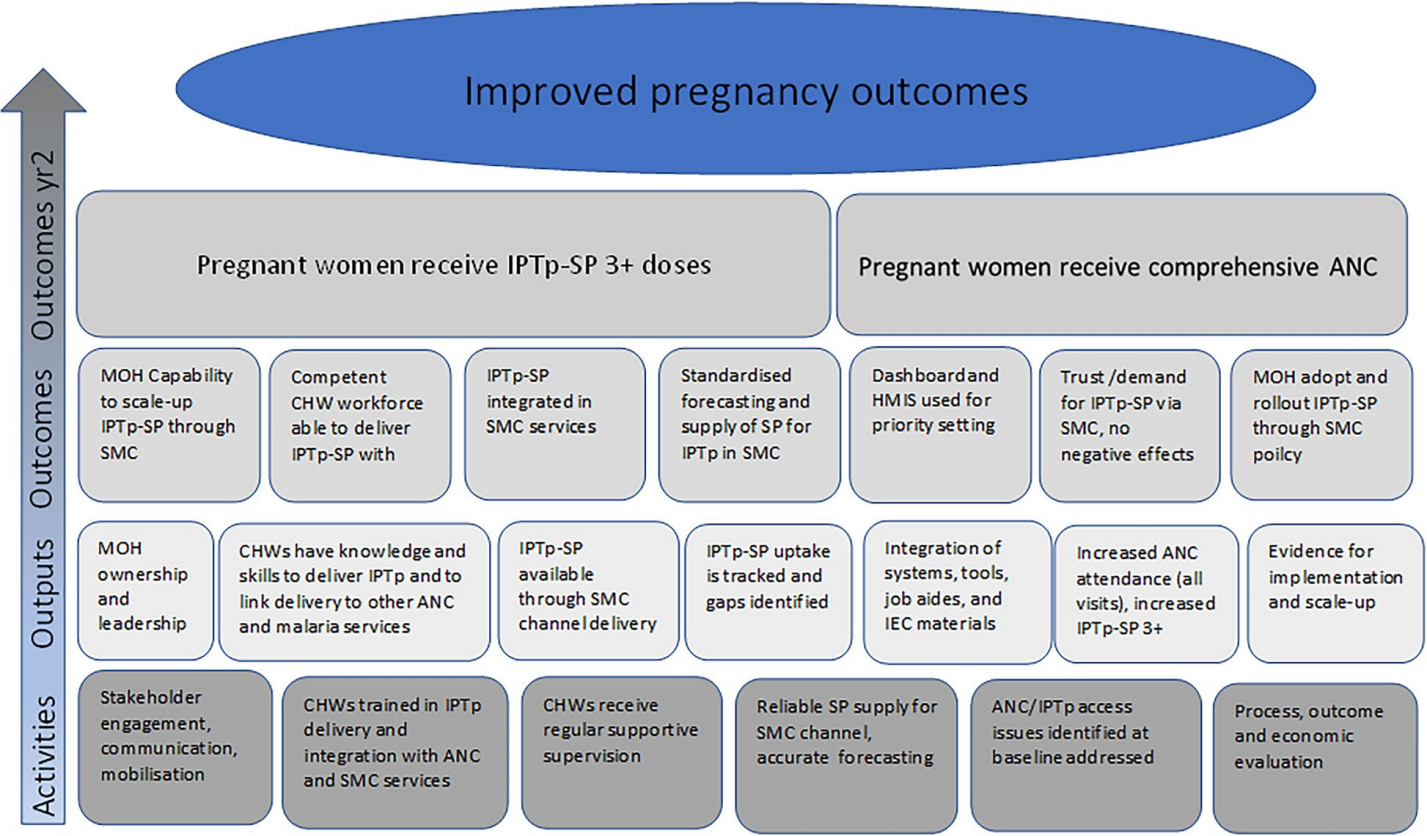
Logic model

Figure 5 summarises the pre-study intervention logic model for the evaluation, describing the proposed causal relationships between the intervention components and outcomes. This model represents the study’s initial overarching conceptual framework to structure data collection and analysis and maintain consistency across countries.

- Clinical outcomes

Individual data on IPTp-SP coverage and ANC attendance will be collected from ANC cards and registers to triangulate with women’s self-reported data from household surveys. These data will be extracted for all ANC clinic attendees during the study period. All registers will be reviewed and data on gestational age and IPTp-SP administration extracted onto electronic study forms previously designed in REDCap, using tablets.

The number of confirmed malaria cases in both children and pregnant women will be recorded from hospital and health centres in both intervention and control arms in all clusters. From the same clusters, pregnancy outcomes from women who deliver in a health facility will be monitored at delivery to record birth weight, preterm delivery, still birth, miscarriage. Table 2 shows the outcomes and their related objectives, methods and timeline.

**Table 2:** Study objectives, outcomes, methods, and timeline

Study objectives	Outcomes	Methods	Timeline
1. To compare the delivery of IPTp-SP through the integrated strategy (ANC + SMC) with standard care (ANC)	The proportion of women who received at least 3 doses of IPTp-SP during pregnancy	• Household survey of women with a child < 12 months	• Baseline • Endline
2. To assess the acceptability and feasibility of ANC + SMC delivery of IPTp-SP compared to standard care (ANC) among health providers, CHWs, and pregnant women and women with a child < 12 months	Major themes emerging through thematic analysis using a theory of acceptability (developed a priori)	• In-depth interviews • Focus group discussions	• Endline
3. To assess the systems effectiveness of the integrated delivery of IPTp-SP	Cumulative systems effectiveness of IPTp-SP delivery at ANC + SMC	• ANC exit interview of ANC attendees	• Endline
4. To assess any increase or decrease in ANC uptake following the integrated ANC + SMC IPTp-SP delivery	The proportion of women with 4 + ANC visits during pregnancy	• Household surveys of women with a child < 12 months • Routine data collection: individual facility data on ANC attendance	• Baseline • Endline • Throughout the trial
5. To assess any increase or decrease in SMC uptake following the integrated ANC + SMC IPTp-SP delivery	The proportion of children 3–59 months who received 4 doses of SMC	• Household surveys of mothers of children 3–59 months	• Baseline • Endline
6. To estimate the number of malaria cases in both children and pregnant women over the course of the trial, by arm	Confirmed malaria • incidence rates and malaria positivity (RDT + /RDT performed) ratios	• Routine data collection: individual facility data on malaria cases for pregnant women and children 3–59 months	• Throughout the trial
7. To estimate the number of poor birth outcomes in women over the course of the trial, by arm	• The proportion of women with miscarriage, stillbirth, low birth weight, or preterm birth	• Routine data collection: individual facility data on birth outcomes	• Throughout the trial
8. To assess care-seeking practices during COVID and the anticipated acceptability of the integrated strategy of ANC + SMC delivery of IPTp-SP	• Major themes emerging through thematic analysis using a theory of acceptability (developed a priori)	• In-depth interviews with healthcare providers • Focus group discussions with women	• Baseline

### Analysis plan

#### Primary endpoints—SMC, IPTp-SP, and ANC analysis

The primary outcome will be the individual level binary indicator for presence or absence of at least three doses of IPTp-SP in women included in the endline IPTp-SP HHS, and the intervention effect will be estimated using a generalized linear mixed model with a logit link function and a random intercept term for cluster – period, expressed as odds ratio and 95% confidence intervals. The primary analysis will be done by country. In a secondary analysis, data of both countries will be combined using an individual patient data meta-analysis approach to assess the overall efficacy of the intervention.

Evolution over time, and by arm, of the use of ANC (number and timing of contacts) will be assessed using routine data and modelling-temporal analysis. In addition, a multilevel logistic regression model will be performed to identify individual and contextual factors associated with ANC attendance and IPTp-SP coverage at baseline. Multilevel modelling will be selected due to the clustering of the collected data.

Differences in SMC coverage (3 or 4 doses) and ANC uptake (4 visits) between control and intervention arms at endline will be assessed using multilevel regression models.

No interim analysis will be conducted.

#### Feasibility and acceptability data analysis

Qualitative data analysis will use a standard thematic procedure. The transcripts will be read and coded by the researchers using the topic guide and the initial coding frame. Conceptual maps will be used to explore relationships and connections in the data and develop specific questions to investigate in further analysis. We will draw on more than one framework. The acceptability theory will draw on Sekhon’s constructs of acceptability [26]. ‘Intervention, actor, context, mechanism and outcome’ configuration [27] will be developed for individual cases and qualitative comparative analysis aiming to identify drivers of acceptability across cases within and across countries. Framework analysis will be used to identify factors influencing the effectiveness of delivery and scalability of each of the interventions using existing frameworks such as the consolidated framework for implementation research [28] and the building blocks of the health system as defined by the WHO [29].

#### Systems effectiveness

Four systems effectiveness analyses will be undertaken: (1) cumulative systems effectiveness of IPTp-SP delivery on the day of the interview i.e., the proportion of women reporting to have received a dose of 3 tablets of SP in the second trimester; (2) effectiveness of each intermediate process in systems effectiveness of IPTp-SP delivery on the day of the visit; (3) effectiveness of IPTp-SP delivery during the woman’s pregnancy i.e., the proportion of women reporting having completed three or more ANC visits and having received three or more doses of SP at four weeks intervals with the first dose given in the second trimester; (4) effectiveness of IPTp-SP in the community i.e., proportion of women having received SP doses in the community within at least four weeks apart from any other doses received from any source. Potential predictors of systems effectiveness will be assessed using univariate and multivariate adjusted logistic regression analyses.

#### Process evaluation

Site, intervention, process and content data will be analyzed descriptively and used to assess whether the study intervention was delivered as intended. When and how much of the intervention was received by pregnant women and any adaptations to the intervention made in each site context will also be captured. In-depth interview data will be analyzed retroductively by at least three investigators to increase the credibility to identify patterns in responses to intervention components within specific contexts that point to possible pathways of causation. Quantitative and qualitative data will be synthesized to explore the three domains of i) implementation through assessment of fidelity, dose, reach and adaptation to understand if the intervention was delivered as intended, and what was modified throughout implementation; ii) context, by identifying factors influencing implementation of the intervention and its outcomes; and iii) mechanisms underscoring how the intervention components were responded to/engaged with to produce changes in practice, experiences and outcomes.

## Discussion

More than 12 million pregnant women are exposed to malaria in SSA, and less than 60% of pregnant women in Mali and Burkina Faso have received IPTp3 + [2]. To increase IPTp3 + coverage and prevent malaria in pregnancy and its consequences, appropriate interventions focused on the period of high malaria transmission are urgently needed [5]. This multicenter cluster randomized implementation trial powered to detect a 45% and 22% increase in IPTp3 + uptake in Mali and Burkina-Faso, respectively, will generate evidence on the feasibility, acceptability, effectiveness, and cost-effectiveness of IPTp-SP delivered through the ANC + SMC channel. To our knowledge this intervention is the first to use the strategy of combining the delivery of IPTp-SP through SMC to increase both the IPTp-SP and ANC uptake. It is designed to facilitate scalability and translation into policy by leveraging existing resources, while strengthening local capacities in research, health, and community institutions. Findings will constitute an evidence base for potential policy changes in malaria prevention in pregnancy, allowing local national malaria control programmes to reach their goals.

## Data Availability

Not applicable.

## Abbreviations

ANC: Antenatal Clinic
AQ: Amodiaquine
CHWs: Community Health Workers
CRs: Community Relays
DE: Design effect
DOT: Direct Observed Treatment
FGDs: Focus group discussions
HF: Health Facility
HHS: Household survey
HMIS: Health Management Information Systems
ICC: Intra Cluster Correlation
IEC: Information, Education and Communication
IPTp: Intermittent preventive treatment during pregnancy
IPTp-SP: Intermittent preventive treatment during pregnancy with Sulfadoxine-pyrimethamine
IPTp3 +: Three or more doses of IPTp-SP
IPTp2 +: Two or more doses of IPTp-SP
MOH: Ministry of Health
PPS: Probability Proportional to Size Sampling
RDT: Rapid Diagnostic Test
SP: Sulfadoxine-Pyrimethamine
SMC: Seasonal Malaria Chemoprevention
WHO: World Health Organization
